# Anthropometric growth trajectories of children presenting with presumptive pulmonary TB

**DOI:** 10.5588/ijtldopen.24.0489

**Published:** 2025-03-12

**Authors:** M.V. de Geus, M. van Niekerk, C. McKenzie, I. Dewandel, J. Zenhausern, E. Wijstma, R. Dunbar, H. Rabie, A.C. Hesseling, V.W. Jongen, M.M. van der Zalm

**Affiliations:** ^1^ Desmond Tutu TB Centre, Department of Paediatrics and Child Health, Faculty of Medicine and Health Sciences, Stellenbosch University, Cape Town, South Africa;; ^2^ Department of Infectious Diseases, Public Health Service Amsterdam, Amsterdam, The Netherlands;; ^3^ Department of Paediatrics, Karl Bremer Hospital, Cape Town, South Africa;; ^4^ Paediatric Infectious Diseases, Department of Paediatrics and Child Health, Faculty of Medicine and Health Sciences, Stellenbosch University, Cape Town, South Africa;; ^5^ Stichting HIV Monitoring, Amsterdam, The Netherlands.

**Keywords:** growth, paediatric, tuberculosis, post-tuberculosis, South Africa

## Abstract

**BACKGROUND:**

This study assessed growth trajectories in children presenting with presumptive pulmonary TB (PTB).

**METHODS:**

This sub-study of the Umoya TB diagnostic study was conducted in South Africa from November 2017 until November 2021. Children (0–13 years) with presumptive PTB were recruited from and followed up for 12 months. Anthropometric measurements of children with TB, symptomatic controls (TB excluded), and healthy controls were taken at baseline and follow-up (2, 8, 16, 24 and 52 weeks). Changes in weight-for-age *Z*-score (WAZ), height-for-age *Z*-score (HAZ) and body mass index for age (BAZ) over time were assessed using multivariable mixed-effect linear regression adjusted for confounders.

**RESULTS:**

Of the 372 children included in the analyses (median age: 2 years, IQR 1–4), 153 children had TB, 168 were symptomatic and 51 were healthy controls. Median WAZ was similar between groups; however, more children with TB were underweight than symptomatic and healthy controls. WAZ increased over time for children with TB. Median HAZ of children with TB (–1.34, IQR –2.17 to –0.21) was lower compared to symptomatic (–1.06, IQR –1.90 to –0.10) and healthy controls (–0.74, IQR –1.26 to –0.03; *P* = 0.0037). There was no significant change over time for HAZ.

**CONCLUSION:**

To improve the long-term outcomes of TB and other illnesses, the overall nutrition of children needs to be improved.

In 2022, an estimated 1.25 million children below the age of 15 years developed TB globally.^1^ There is increasing evidence that, even after successful treatment, TB disease can have lasting consequences.^2^ Although these data mostly pertain to adults, recent studies demonstrated similar long-term effects of TB on health and quality of life in children and adolescents.^3–5^ In addition, a recent birth-cohort study found lower height-for-age (HAZ) and weight-for-age *Z*-scores (WAZ) in children at the age of 5 years if they had TB in the first 5 years of life compared to children who did not have TB.^4^ This is a concern because early-life growth is crucial to prevent stunting, avert lasting neurodevelopmental impairment, and ensure normal lung growth.^6,7^

Nutrition and growth are closely related, as sufficient nutritional intake is essential for optimal growth.^8^ Height faltering is associated with chronic undernutrition, other reasons like chronic illnesses, and constitutional and socio-economic factors.^8^ Impaired growth in height and weight is also associated with higher risks of mortality and morbidity, partially due to the association of malnutrition with compromised immune function, making malnourished children more vulnerable to diseases.^9,10^ In addition to the increased metabolism resulting from illness, TB can cause a lack of appetite and subsequent acute weight loss or growth failure.^11,12^ This results in a vicious cycle where children with TB are more vulnerable to undernutrition, and children with undernutrition are more susceptible to disease.^13^ It is estimated that in the 30 countries with the highest TB burden, 24.1% of TB cases between 2016 and 2020 were attributed to undernutrition.^14^ The relationship between nutrition and TB was further stressed by a recent randomised controlled trial that showed a relative reduction of 39–48% in the incidence rate of pulmonary TB (PTB) over a 2-year period after supplementing with macro- and micronutrients among household contacts of patients with TB.^15^

Limited data are available on the growth of children with TB and how their growth trajectories after completing TB treatment compared to children without TB. Therefore, this study assessed the growth trajectories for weight and height in South African children aged 0–13 years with presumptive PTB during a 12-month follow-up period, including children eventually treated for TB, symptomatic children in whom TB was excluded and healthy controls.

## METHODS

### Study design and population

The Umoya study is an ongoing prospective cohort study based in Cape Town, South Africa, which focuses on TB diagnosis and the long-term impact of PTB. Methods have been described previously.^16^ In summary, children aged 0–13 years were recruited between 23 November 2017, until now from Karl Bremer Hospital (KBH, a secondary-level hospital) and Tygerberg Hospital (TBH, a secondary and tertiary-level hospital) in Cape Town, South Africa. Children with presumptive PTB (who were started on TB treatment, both confirmed and unconfirmed) and symptomatic controls (children with symptoms similar to TB but TB diagnosis excluded after careful investigation and follow-up) were enrolled. Healthy siblings of children with presumptive PTB (referred to as ‘healthy controls’ hereafter) were not matched by age or sex but were included to provide a local comparison group with similar socio-economic backgrounds. Exclusion criteria for enrolment included receiving TB treatment for more than 2 days in the 2 weeks before inclusion, solely extrathoracic TB with no signs of PTB, inability to attend follow-up visits, and other severe illness limiting the ability to collect respiratory TB samples. For the current study, only children between 23 November 2017 and 17 November 2021, were included to ensure all children were in the cohort for at least 52 weeks.

### Classification of TB disease

The investigations for TB included a combination of Tuberculin Skin Test (TST) and *Mycobacterial tuberculosis* (MTB) microbiology on at least two respiratory samples (gastric aspirate, induced sputum, or expectorated sputum) including concentrated acid-fast bacilli smear microscopy, TB culture (BACTEC MGIT™ 960™ liquid medium; BD, Franklin Lakes, NJ, USA) and Xpert^®^ MTB/RIF (Cepheid, Sunnyvale, CA, USA) until March 2018 or Ultra (Cepheid) from March 2018 on all respiratory samples. Positive cultures were further tested for drug susceptibility using a line probe assay.

Children with TB were classified as confirmed TB if microbiological confirmation was positive on any respiratory sample (culture or Xpert MTB/RIF or Ultra) and unconfirmed TB if there was no microbiological confirmation. The treating physicians decided whether to start TB treatment based on clinical presentation, all available microbiology results and radiological images. Children who presented with TB-related symptoms but who were not started on TB treatment after careful examination and follow-up were included as symptomatic controls.

### Procedures

Sociodemographic characteristics and clinical background were recorded at baseline. Anthropometric data (weight and height) were collected at baseline and during follow-up (2, 8, 16, 24, and 52 weeks after enrolment) using standardised procedures. In some cases, the instruments used at baseline differed from those used during follow-up, but subsequently, the instruments remained consistent. For children 0–2 years, weight was measured using a pediatric electronic scale and length using a length board. For children older than 2 years, a standing electronic scale was used for weight and a stadiometer for height. HAZ, WAZ and BMI-for-age (BAZ) were calculated using the *zscore06* (for children up to 5 years of age) and *zanthro* (for children 5–10 years of age) plug-ins for STATA (StataCorp, College Station, TX, USA; 2021)^17,18^ based on the WHO and the Centers for Disease Control and Prevention (CDC) standards, designed to assess children’s growth by comparison to child growth under optimal circumstances.^19^ Underweight is defined as WAZ <–2; stunting as HAZ <–2; wasting as BAZ of <–2.^20^

### Statistical analysis

Children were included if they had at least two measurements for weight and height (i.e., at baseline and at least one follow-up visit). Continuous variables were reported by mean and standard deviation (SD), or median and interquartile range (IQR) in case of a non-normal distribution and compared using *t*-tests, Kruskal-Wallis or Wilcoxon rank-sum tests. Categorical variables were reported using frequencies and percentages and compared using Pearson’s χ^2^ test or Fisher’s exact test.

Three mixed-effect linear regression models were used to analyse the longitudinal effect of time on WAZ, HAZ and BAZ for the three study arms. We included a random intercept to correct for within-participant correlation. Time and study arm were included in the model, and an interaction term between time and study arm was used in all three models. Possible confounders (sex, birth weight and length, maternal smoking during pregnancy, and pre-term birth) were included in multivariable models. To assess the potential bias caused by participants not attending the Week 52 visit, we repeated the crude analyses on a subset of participants who attended the Week 52 visit.

We used a logistic regression model with a clustered variance-covariance matrix to assess the effect of time on being underweight or stunted for the three study arms. We added time and study arm to the model and an interaction term between time and study arm. Data analysis was performed using Stata. A *P* < 0.05 was considered statistically significant.

### Ethical approval

Ethical approval was provided by the Health Research Ethics Committee of Stellenbosch University, Cape Town, South Africa (N17/08/083). All parents/legal guardians provided written informed consent before enrolment in the study; children ≥7 years also provided written assent.

## RESULTS

Between 23 November 2017 and 17 November 2021, 400 children were enrolled in the Umoya study, of whom 372 (93%) were included in the analyses. Children were excluded due to withdrawal of consent (*n* = 13) or loss to follow-up after enrolment (*n* = 15). In total, 153 (41.0%) children with TB (both confirmed and unconfirmed) were included, 168 (45.3%) symptomatic controls, and 51 (13.7%) healthy controls. Children with TB were younger (median: 1 year, IQR 0–3) than symptomatic controls (median: 2 years, IQR 1–4) and healthy controls (median: 4 years, IQR 3–8; *P* < 0.001) (Table 1). Of the 372 children, 27 (7.2%) children were living with HIV and 90 (24.9%) children were perinatally exposed to HIV. Most (81.6%) of children came from families that received financial support from the South African government. Hundred and fifty-four mothers smoked during pregnancy, and 72 (21%) children were born pre-term (<37 weeks), more children with TB were born premature.

**Table 1.** Baseline socio-demographic and clinical characteristics of the participants included in these analyses, November 2017–November 2021, Cape Town, South Africa.

	Total (*n* = 372) *n* (%)	Children with TB (*n* = 153) *n* (%)	Symptomatic controls (*n* = 168) *n* (%)	Healthy controls (*n* = 51) *n* (%)	*P*-value^*^
Sociodemographics					
Age, years, median [IQR]	2 [1–4]	1 [0–3]	2 [1–4]	4 [3–8]	<0.001
Age groups, years					<0.001
0–1	166 (45)	86 (56)	76 (45)	4 (8)	
2–5	193 (37)	50 (33)	61 (36)	28 (55)	
≥6	67 (18)	17 (11)	31 (18)	19 (37)	
Sex					0.331
Male	192 (52)	73 (48)	94 (56)	25 (49)	
Female	180 (48)	80 (52)	74 (44)	26 (51)	
Ethnicity					0.053
Mixed ancestry	221 (59)	78 (51)	115 (68)	28 (55)	
Black	147 (40)	72 (47)	52 (31)	23 (45)	
Other	4 (1)	3 (2)	1 (0.6)	0 (0)	
Household size					0.120
<6 persons	147 (40)	66 (43)	57 (34)	24 (47)	
≥6 persons	225 (60)	87 (57)	111 (66)	27 (53)	
Type of housing^†^					0.133
Formal	249 (67)	100 (65)	120 (71)	29 (57)	
Informal	123 (33)	53 (35)	48 (29)	22 (43)	
Cooking on paraffin stove					0.368
No	360 (97)	147 (96)	162 (96)	51 (100)	
Yes	12 (3)	6 (4)	6 (4)	0 (0)	
Primary caregiver					0.653
Parent	321 (86)	135 (88)	143 (85)	43 (84)	
Other	51 (14)	18 (12)	25 (15)	8 (16)	
Any grants received^‡^					0.652
No	68 (18)	29 (19)	32 (19)	7 (14)	
Yes	302 (82)	123 (81)	135 (81)	44 (86)	
Missing	2	1	1	0	
Early childhood					
Maternal smoking during pregnancy					0.644
No	213 (58)	92 (61)	94 (56)	27 (56)	
Yes	154 (42)	59 (39)	74 (44)	21 (44)	
Missing	5	2	0	3	
Household smoking after birth					0.550
No	144 (39)	64 (42)	63 (38)	17 (34)	
Yes	227 (61)	89 (58)	105 (62)	33 (66)	
Missing	1	0	0	1	
Pre-term birth (<37 weeks)					0.034
No	273 (80)	102 (72)	134 (84)	37 (82)	
Yes^§^	72 (21)	39 (28)	25 (16)	8 (18)	
Missing	27	12	9	6	
Birth weight, g, median [IQR]^¶^	2,910 [2,570–3,290]	2,878 [2,451–3,260]	3,000 [2,596–3,340]	2,865 [2,605–3,190]	0.201
Low birth weight (<2,500 g)					0.308
No	261 (78)	104 (74)	121 (80)	36 (84)	
Yes	73 (22)	36 (26)	30 (20)	7 (16)	
Missing	38	13	17	8	
Birth length, cm, median [IQR]^#^	49 [47–51]	49 [46–51]	49 [47–52]	48 [46–50]	0.150
Received BCG vaccine					0.006
No	29 (8)	20 (13)	6 (4)	3 (6)	
Yes	343 (92)	133 (87)	162 (96)	48 (94)	
Type of feeding					0.138
Exclusive breastfeeding	268 (74)	114 (76)	124 (76)	30 (64)	
Exclusive bottle	60 (17)	23 (15)	23 (14)	14 (30)	
Mixed	33 (9)	14 (9)	16 (10)	3 (6)	
Missing	11	2	5	4	
Duration of exclusive breastfeeding, months, median [IQR]^**^	6 [3–6]	6 [3–6]	5 [3–6]	6 [3–6]	0.256
Timing of solid food initiation, months, median [IQR]^††^	6 [4–6]	6 [4–6]	6 [4–6]	6 [4–6]	0.664
Health-related					
TST-positive					NA
No	38 (53)	38 (54)	0 (0)	NA	
Yes	34 (47)	32 (46)	2 (100)	NA	
Missing	300	83	166	NA	
Recent deworming^‡‡^					0.052
No	113 (45)	39 (38)	52 (46)	22 (61)	
Yes	140 (55)	64 (62)	62 (54)	14 (39)	
Missing	45	14	19	12	
Child living with HIV					0.065
No	345 (93)	138 (90)	156 (93)	51 (100)	
Yes	27 (7)	15 (10)	12 (7)	0 (0)	
HIV-exposed					0.109
No	271 (75)	105 (70)	130 (80)	36 (77)	
Yes	90 (25)	46 (30)	33 (20)	11 (23)	
Missing	11	2	5	4	
Previous TB treatment					0.633
No	339 (91)	142 (93)	151 (90)	46 (90)	
Yes	33 (9)	11 (7)	17 (10)	5 (10)	

* Calculated using χ^2^ and Kruskal Wallis tests to assess the differences between the three study arms.

† Includes brick house, informal housing includes wendy house or shack.

‡ Any type of financial support from the South African government.

§ 15 children were born between 28–31 weeks and 49 between 32–37 weeks. For 8 children who were born prematurely, the exact gestational age was unknown.

¶ 38 missing.

# 96 missing.

** Among those who were breastfed (*n* = 250).

†† 48 missing.

‡‡ Among children >12 months of age (*n* = 298).

IQR = interquartile range, BCG = bacille Calmette-Guérin, TST = tuberculin skin test, NA = not applicable.

Due to the COVID-19 pandemic, 248 (66.7%) children were unable to attend the week-52 follow-up visit. The median follow-up time was 25.6 weeks (IQR 23.6–42.8). Among the 372 participants, a total of 1,892 measurements for weight and height were available over time. In total, 119 children had six or more weight and height measurements, 163 children had five, 41 children had four, 31 children had three, and 18 children had two.

### Weight-for-age

**Figure 1.**
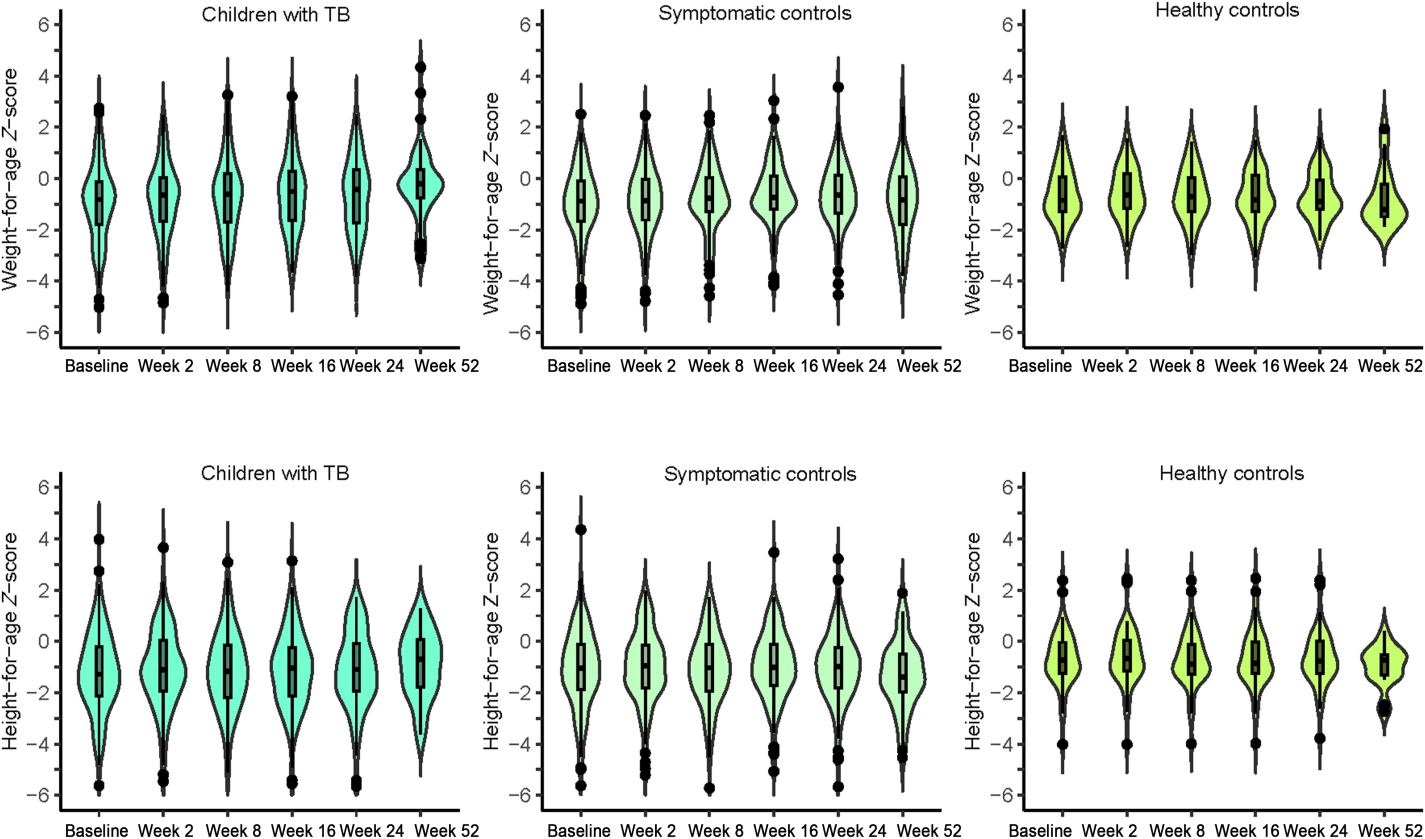
Median weight-for-age *Z*-score and height-for-age *Z*-score at baseline and follow-up visits per study arm. The boxplot indicates the interquartile range with the median represented by the black line and the shape around the boxplot represents the distribution of the data. The dots represent outliers within the data.

Overall, WAZ was –0.87 (IQR –1.68 to –0.07) at baseline; –0.84 (IQR –1.88 to –0.12) for children with TB, –0.88 (IQR –1.68 to –0.08) for symptomatic controls, and –0.85 (IQR –1.30 to 0.08) for healthy controls (*P* = 0.483, Figure 1, Supplementary Table S2). After 52 weeks of follow-up, the overall median WAZ was –0.58 (IQR –1.39 to 0.10), and WAZ increased to –0.21 (IQR –0.78 to 0.38) for children with TB. At baseline, children with TB were more often underweight (*n* = 34, 22%) than symptomatic controls (*n* = 28, 17%) and healthy controls (*n* = 3, 6%) (*P* = 0.027) (Table 2). Follow-up assessments revealed that children with TB were also more often underweight at later study visits compared to symptomatic and healthy controls, albeit only statistically significant at week 24 (*P* = 0.047) (Table 2).

**Table 2.** Proportions of children underweight^*^ and with stunting^†^ per study arm at baseline, Weeks 2, 8, 16, 24 and 52.

	Children with TB		Symptomatic controls		Healthy controls		
	*N*	*n* (%)	*N*	*n* (%)	*N*	*n* (%)	*P*-value^‡^
Baseline							
Underweight	153	34 (22)	168	28 (17)	51	3 (6)	0.027
Stunting	153	45 (30)	168	39 (23)	51	4 (8)	0.007
Week 2							
Underweight	150	30 (20)	162	27 (17)	51	4 (8)	0.134
Stunting	150	36 (24)	162	29 (18)	51	4 (8)	0.035
Week 8							
Underweight	137	30 (21)	142	21 (15)	47	4 (9)	0.072
Stunting	137	39 (28)	142	30 (21)	47	5 (11)	0.035
Week 16							
Underweight	126	22 (17)	132	11 (8)	43	6 (14)	0.090
Stunting	126	38 (30)	132	24 (18)	43	5 (12)	0.013
Week 24							
Underweight	107	21 (20)	124	13 (10)	44	3 (7)	0.047
Stunting	107	26 (24)	124	25 (20)	44	5 (11)	0.200
Week 52							
Underweight	46	6 (13)	62	12 (19)	16	0 (0)	0.146
Stunting	46	9 (20)	62	14 (23)	16	2 (13)	0.664

* Defined as a weight-for-age *Z*-score <–2.

† Defined as a height-for-age *Z*-score <–2.

‡ Calculated using Pearson’s χ^2^ or Fisher’s exact tests to assess differences between the three study arms.

**Figure 2.**
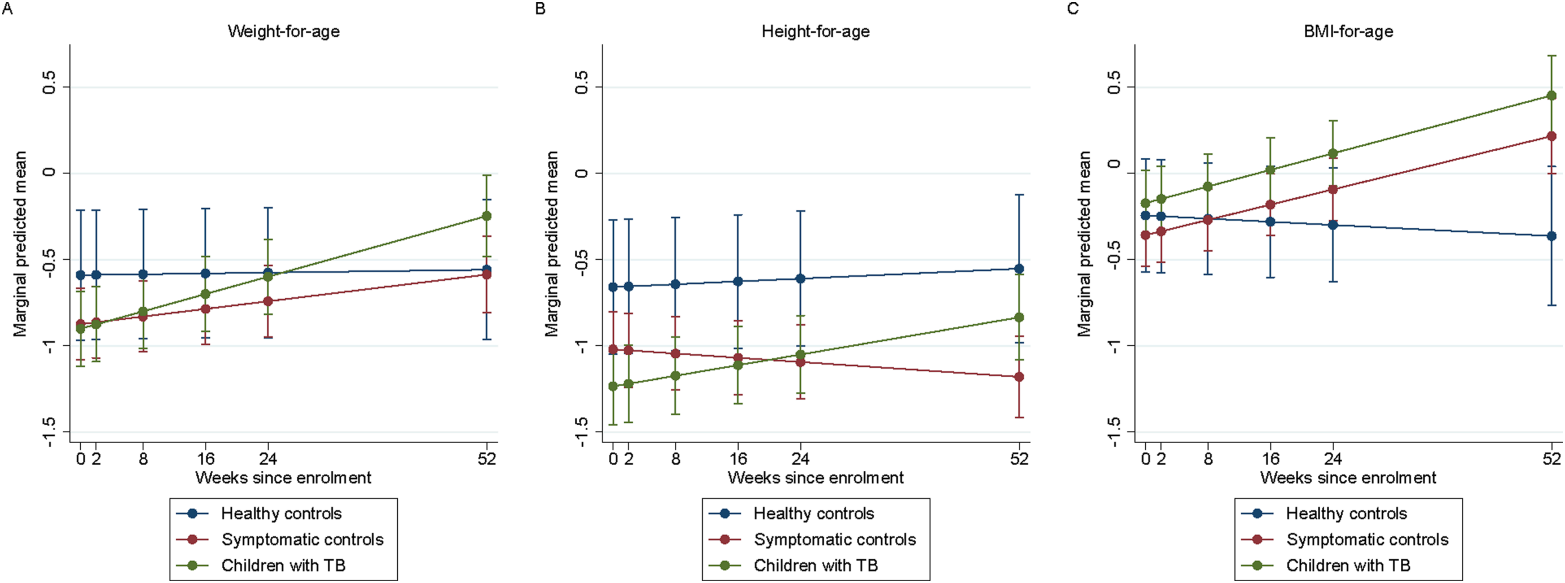
Predicted mean WAZ score and HAZ score for each study arm. **A)** WAZ over time based on the unadjusted mixed-effect linear model. The marginal trend of WAZ score improves over time for children with TB and symptomatic controls and gets back on the same trajectory of healthy controls. **B)** HAZ over time based on the unadjusted mixed-effect linear model. The marginal trend of HAZ score for children with TB and symptomatic controls is below the trend of the healthy controls but still increases over time. **C)** BAZ over time based on the unadjusted mixed-effect linear model. The marginal trend of BAZ score score improves over time for children with TB and symptomatic controls. WAZ = weight-for-age *Z*; HAZ = height-for-age *Z*; BAZ = BMI-for-age *Z*; BMI = body mass index.

In the multivariable analyses only including study arm and time, WAZ slightly increased over time for children with TB (β = 0.012 per week increase, 95% CI 0.007 to 0.017; *P* < 0.001) and symptomatic controls (β = 0.005 per week increase, 95% CI 0.0005 to 0.009; *P* = 0.030), than healthy controls (Figure 2, Supplementary Table S2). After adjusting for sex, birth weight and length, maternal smoking during pregnancy, and pre-term birth, WAZ significantly increased over time for children with TB (adjusted β = 0.011 per week increase, 95% CI 0.005 to 0.018; *P* = 0.001), but not for symptomatic controls (adjusted β = 0.002 per week increase, 95% CI –0.004 to 0.009; *P* = 0.493) (Table 3). In the sensitivity analysis, participants who attended the Week 52 visit (*n* = 124) were only included, and the results were similar, albeit the increase in WAZ for symptomatic controls was no longer statistically significant (Supplementary Table S3). Over time, the odds of being underweight did not change for children with TB (OR 1.003 per week increase, 95% CI 0.988 to 1.02; *P* = 0.635) or symptomatic controls (OR 1.00 per week increase, 95% CI 0.987 to 1.01; *P* = 0.989), than healthy controls.

**Table 3.** Adjusted mixed-effect linear models for WAZ and HAZ in children with TB, symptomatic controls and healthy controls.^*^

	Coefficient (β)	95% CI	*P* value
WAZ score			
Time, weeks	0.004	–0.002 to 0.010	0.192
Study arm			
Healthy controls	Reference	—	—
Symptomatic controls	–0.211	–0.718 to 0.296	0.415
Children with TB	–0.271	–0.779 to 0.229	0.237
Study arm interaction with time, weeks			
Healthy controls	Reference	—	—
Symptomatic controls	0.002	–0.004 to 0.009	0.493
Children with TB	0.011	0.005 to 0.018	0.001
HAZ score			
Time, weeks	0.002	–0.006 to 0.010	0.596
Study arm			
Healthy controls	Reference	—	—
Symptomatic controls	–0.436	–0.965 to 0.093	0.106
Children with TB	–0.484	–1.014 to 0.046	0.073
Study arm interaction with time, weeks			
Healthy controls	Reference	—	—
Symptomatic controls	–0.006	–0.014 to 0.002	0.150
Children with TB	0.007	–0.001 to 0.016	0.088
BMI-for-age *Z*-score			
Time, weeks	–0.0004	–0.010 to 0.009	0.925
Study arm			
Healthy controls	Reference	—	—
Symptomatic controls	0.060	–0.388 to 0.508	0.793
Children with TB	0.143	–0.306 to 0.592	0.532
Study arm interaction with time, weeks			
Healthy controls	Reference	—	—
Symptomatic controls	0.013	0.004 to 0.023	0.008
Children with TB	0.014	0.004 to 0.024	0.006

* Models were adjusted for sex, birth weight and length, maternal smoking during pregnancy and pre-term birth.

WAZ = weight-for-age *Z*; HAZ = height-for-age *Z*; CI = confidence interval; BMI = body mass index.

### Height-for-age

Overall, median HAZ was –1.06 (IQR –1.96 to –0.13) at baseline; median HAZ of children with TB (median –1.34, IQR –2.17 to –0.21) was lower than symptomatic controls (median –1.06, IQR –1.90 to –0.10) and healthy controls (median –0.74, IQR –1.26 to –0.03; *P* = 0.037) (Figure 1, Supplementary Table S1). At subsequent study visits, the overall median HAZ was similar between all three study arms. However, children with TB had a significantly lower median HAZ at baseline and Weeks 2 and 8 compared to the healthy controls (Supplementary Table S1). Furthermore, 45 (30%) children with TB were stunted at baseline, compared to 39 (23%) symptomatic controls and 4 (8%) healthy controls (*P* = 0.007) (Table 2). Follow-up assessments revealed that stunting remained common over time in all three study arms (Table 2).

In the crude model, HAZ slightly increased over time for children with TB compared to healthy controls (β = 0.006 per week increase, 95% CI 0.0003 to 0.011; *P* < 0.039) (Figure 2, Supplementary Table S2). In adjusted analyses, there was no statistically significant increase over time (β = 0.007 per week increase, 95% CI –0.001 to 0.016; *P* = 0.088) (Table 3). Similarly, in the sensitivity analysis, HAZ no longer significantly increased over time (Supplementary Table S3). Over time, the odds of being stunted did not improve for children with TB (OR 0.98 per week increase, 95% CI 0.969 to 1.01; *P* = 0.182) and symptomatic controls (OR 0.99 per week increase, 95% CI 0.967 to 1.01; *P* = 0.183), than healthy controls.

### BMI-for-age

Overall, BAZ was –0.20 (IQR –1.12 to 0.49) at baseline; –0.18 (IQR –1.24 to 0.63) for children with TB, –0.20 (IQR –0.97 to 0.34) for symptomatic controls, and –0.26 (IQR –0.81 to 0.034) for healthy controls (*P* = 0.908). At baseline, 18 (12%) children with TB, 20 (12%) symptomatic controls, and 3 (6%) healthy controls were wasted (BAZ <–2.0). At subsequent study visits, the median BAZ was similar between all three study arms. In the crude model, BAZ slightly improved over time for children with TB and symptomatic controls compared to healthy controls (Figure 2, Supplementary Table S2). This was similar in the sensitivity analysis among participants who attended the Week 52 visit (Supplementary Table S3).

## DISCUSSION

To our knowledge, this is the first study to study the trajectory of weight, height, and BMI in children with presumptive TB after TB treatment completion and/or following a 12-month follow-up. Our data show a significant increase of WAZ over time in children with TB compared to healthy controls, but the proportion of underweight children remained stable. Weight gain after starting treatment is expected and can be used as a marker for successful treatment response.^21,22^

In terms of HAZ, children with TB had a lower HAZ at baseline compared to healthy controls, and HAZ did not significantly improve over time. Moreover, stunting was present in 20-30% of children with TB and symptomatic controls. The poor long-term nutritional status, as indicated by low HAZ at baseline, potentially made them more susceptible to disease, both TB as well as other infectious diseases.^23^

Pre-natal and early-life nutrition are critical periods for lung development, and growth distortion can permanently affect the structure and function.^7,24^ Data from a large cohort study from Bangladesh suggests that stunted children who experience catch-up growth over time in height have similar lung function to non-stunted children.^25^ However, children with persistent stunting have a reduction of lung function as pre-adolescents. This indicates the importance of normalisation of growth trajectories after disease and suggests an optimal time for targeted nutritional interventions. Our data showed that children presenting with similar symptoms but in whom TB was excluded followed similar growth trajectories to children with TB. Overall, this might indicate that growth trajectories are possibly determined by sociodemographics rather than TB disease in general and nutritional interventions will be important for this vulnerable population.

In our study, HAZ did not show significant improvement over time, which was not unexpected as it is expected to take longer to recover. Other studies investigating long-term growth have observed that children with malnutrition require several years to return to the normal growth trajectory.^26–28^ It will be crucial to observe whether the height trajectories improve and normalise over an extended period in order to thoroughly assess the long-term impact of TB disease on lung health.

Most families in this cohort received government aid, lived in informal housing (i.e., settlements which usually include houses made of iron sheets or individually constructed), and shared their living space with many people, indicating overall low socio-economic circumstances of the study population. Although the median values of WAZ and HAZ were within normal ranges (between –2 and +2 *Z*-score^19^), up to a fifth of children were underweight or stunted. In other parts of the world, improved nutritional status and living conditions were correlated with reductions in TB, even in the absence of TB treatment or vaccination.^29^ We therefore call for interventions that address undernutrition specifically and poverty more broadly in TB-affected communities.

One of the strengths of the study was the inclusion of healthy and symptomatic controls from similar socio-economic backgrounds to compare growth in our setting. Therefore, the differences seen in WAZ and HAZ at baseline uncover the vulnerability of this group of children. The study also has limitations. First, the mixed-effect linear models were adjusted for several confounding factors that are known to have an impact on anthropometric measurements.^6,30^ However, weight and height may be affected by other factors, including the height of parents and maternal diabetes.^31,32^

Furthermore, healthy controls were generally older, and although anthropometric measures were corrected for age and sex, residual confounding might have occurred. Second, the follow-up duration was restricted to one year, and longer observation periods may be necessary to observe height recovery adequately. Third, due to the COVID-19 pandemic, two-thirds of the study population did not attend the Week 52 visit; this resulted in substantial missing data. While we included a random intercept in our models to adjust for potential baseline differences, this did not adjust for any bias caused by loss to follow-up. Additionally, given that the missing visits were non-random, multiple imputation was not possible. Lastly, nutritional intake or blood tests for nutritional status could provide further insights into the relationship between growth, nutrition and disease.

## Supplementary Material


