# Supplementary material for: Anthropometric growth trajectories of children presenting with presumptive pulmonary TB
Source: IJTLD Open. 2025 Mar 12;2(3):137–44. doi: 10.5588/ijtldopen.24.0489 (PMC11906025; doi:10.5588/ijtldopen.24.0489)
Supplement: Supplementary file 1 [file ijtldopen24-0489_supplementarydata1.docx]

# Anthropometric growth trajectories of children presenting with presumptive pulmonary TB

#

**SUPPLEMENTARY DATA**

| Supplementary Table 1. Weight-for-age Z-score (WAZ), height-for-age Z-score (HAZ) and BMI-for-age (BAZ) per study arm at baseline, week 2, 8, 16, 24 and 52 | | | | | | | | |
| --- | --- | --- | --- | --- | --- | --- | --- | --- |
|  | **Children with TB** | | **Symptomatic controls** | | **Healthy controls** | |  |  |
|  | N | Median [IQR] | N | Median [IQR] | N | Median [IQR] | Overall  p-value^a^ | p-value^b^  TB/HC |
| Baseline  WAZ  HAZ  BAZ | 153 153  153 | -0.84 (-1.88;-0.12)  -1.34 (-2.17;-0.21)  -0.18 (-1.24;0.63) | 168 168  168 | -0.88 (-1.68;-0.08)  -1.06 (-1.90;-0.10)  -0.20 (-0.97;0.34) | 51  51  51 | -0.85 (-1.30;0.08)  -0.74 (-1.26;0.03)  -0.26 (-0.81;0.34) | 0.483  0.037  0.908 | 0.271  0.011  0.987 |
| Week 2  WAZ  HAZ  BAZ | 150 150  150 | -0.64 (-1.70;0.04)  -1.14 (-1.97;0.03)  -0.07 (-1.21;0.71) | 162 162  162 | -0.88 (-1.62;-0.04)  -0.96 (-1.81;-0.13)  -0.34 (-1.17;0.40) | 51 51  51 | -0.66 (-1.17;0.19)  -0.67 (-1.16;0.08)  -0.14 (-0.86;0.39) | 0.414  0.058  0.298 | 0.273  0.021  0.712 |
| Week 8  WAZ  HAZ  BAZ | 137 137  137 | -0.64 (-1.71;0.18)  -1.19 (-2.23;-0.18)  -0.09 (-0.96;0.96) | 142 142  142 | -0.78 (-1.30;0.05)  -1.03 (-1.94;-0.10)  -0.11 (-0.92;0.51) | 47 47  47 | -0.70 (-1.28;0.07)  -0.88 (-1.27;-0.08)  -0.08 (-0.76;0.34) | 0.924  0.101  0.403 | 0.732  0.032  0.278 |
| Week 16  WAZ  HAZ  BAZ | 126 126  126 | -0.51 (-1.67;0.31)  -1.04 (-2.14;-0.22)  0.10 (-0.80;1.08) | 132 132  132 | -0.74 (-1.21;0.10)  -1.02 (-1.71;-0.12)  0.02 (-0.60;0.43) | 43 43  43 | -0.84 (-1.31;0.12)  -0.85 (-1.27;0.00)  -0.25 (-0.92;0.39) | 0.791  0.205  0.114 | 0.773  0.094  0.039 |
| Week 24  WAZ  HAZ  BAZ | 107 107  107 | -0.44 (-1.72;0.39)  -1.09 (-1.99;-0.06)  0.10 (-0.75;0.99) | 124 124  124 | -0.65 (-1.36;0.13)  -0.97 (-1.82;-0.23)  -0.06 (-0.78;0.49) | 44 44  44 | -0.91 (-1.21;-0.00)  -0.75 (-1.24;0.03)  -0.29 (-0.95;0.46) | 0.927  0.186  0.090 | 0.759  0.076  0.030 |
| Week 52  WAZ  HAZ  BAZ | 46 46  46 | -0.21 (-0.78;0.38)  -0.68 (-1.79;0.10)  0.38 (-0.58;1.02) | 62 62  62 | -0.84 (-1.79;0.07)  -1.40 (-1.99;-0.48)  0.02 (-0.74;0.59) | 16 16  16 | -1.22 (-1.50;-0.18)  -0.73 (-1.31;-0.49)  -0.67 (-1.20;0.44) | 0.064  0.150  0.124 | 0.056  0.694  0.077 |
| Notes: ^a^ p-value calculated using the Kruskal Wallis tests to assess the differences between the three study arms.  ^b^ p-value calculated using the Wilcoxon rank-sum test to assess the difference between children with TB and healthy controls    Abbreviations: IQR = Interquartile range, TB = tuberculosis, HC = Healthy controls, WAZ = Weight-for-age z-score, HAZ = Height-for-age z-score, BAZ = BMI-for-age z-score | | | | | | | | |

| ***Supplementary Table 2. Crude mixed-effect linear models for WAZ and HAZ in children with TB, symptomatic controls and healthy controls.*** | | | |
| --- | --- | --- | --- |
|  | Coefficient (β) | 95% CI | p-value ^a^ |
| ***Weight-for-age z-score*** | | | |
| Time (weeks) | 0.0006 | -0.003 – 0.005 | 0.768 |
| *Study arm* | | | |
| Healthy controls | REF |  |  |
| Symptomatic controls | -0.285 | -0.715 – 0.145 | 0.194 |
| Children with TB | -0.312 | -0.747 – 0.196 | 0.123 |
| *Study arm interaction with time (weeks)* | | | |
| Healthy controls | REF |  |  |
| Symptomatic controls | 0.005 | 0.0005 – 0.009 | 0.030 |
| Children with TB | 0.012 | 0.007 – 0.017 | <0.001 |
|  | | | |
| ***Height-for-age z-score*** | | | |
| Time (weeks) | 0.002 | -0.003 – 0.007 | 0.386 |
| *Study arm* | | | |
| Healthy controls | REF | - | - |
| Symptomatic controls | -0.361 | -0.807 – 0.085 | 0.113 |
| Children with TB | -0.576 | -1.028 – -0.125 | 0.012 |
| *Study arm interaction with time (weeks)* | | | |
| Healthy controls | REF | - | - |
| Symptomatic controls | -0.005 | -0.010 – 0.0001 | 0.056 |
| Children with TB | 0.006 | 0.0003 – 0.011 | 0.039 |
|  |  |  |  |
| ***BMI-for-age z-score*** |  |  |  |
| Time (weeks) | -0.002 | -0.008 – 0.004 | 0.453 |
| *Study arm* |  |  |  |
| Healthy controls | REF | - | - |
| Symptomatic controls | -0.114 | -0.490 – 0.262 | 0.551 |
| Children with TB | 0.072 | -0.308 – -0.452 | 0.711 |
| *Study arm interaction with time (weeks)* | | | |
| Healthy controls | REF | - | - |
| Symptomatic controls | 0.013 | 0.007– 0.020 | <0.001 |
| Children with TB | 0.014 | 0.007 – 0.021 | <0.001 |
| **Abbreviations:** 95% CI = 95% confidence interval, TB = tuberculosis, WAZ = weight-for-age z-score, HAZ = height-for-age z-score | | | |

| ***Supplementary Table 3.*  Sensitivity analysis among participants who attended their week 52 visit (n=124). Crude mixed-effect linear models for WAZ and HAZ in children with TB, symptomatic controls and healthy controls.** | | | |
| --- | --- | --- | --- |
|  | Coefficient (β) | 95% CI | p-value ^a^ |
| ***Weight-for-age z-score*** | | | |
| Time (weeks) | -0.0002 | -0.005 – 0.005 | 0.948 |
| *Study arm* | | | |
| Healthy controls | REF |  |  |
| Symptomatic controls | -0.203 | -0.923 – 0.516 | 0.579 |
| Children with TB | 0.149 | -0.595 – 0.894 | 0.694 |
| *Study arm interaction with time (weeks)* | | | |
| Healthy controls | REF |  |  |
| Symptomatic controls | 0.004 | -0.001 – 0.010 | 0.123 |
| Children with TB | 0.009 | 0.003 – 0.015 | 0.003 |
|  | | | |
| ***Height-for-age z-score*** | | | |
| Time (weeks) | 0.002 | -0.005 – 0.009 | 0.600 |
| *Study arm* | | | |
| Healthy controls | REF | - | - |
| Symptomatic controls | -0.091 | -0.832 – 0.649 | 0.809 |
| Children with TB | -0.019 | -0.785– 0.748 | 0.962 |
| *Study arm interaction with time (weeks)* | | | |
| Healthy controls | REF | - | - |
| Symptomatic controls | -0.006 | -0.014 – 0.001 | 0.110 |
| Children with TB | 0.005 | -0.003 – 0.013 | 0.189 |
|  |  |  |  |
| ***BMI-for-age z-score*** |  |  |  |
| Time (weeks) | -0.002 | -0.010 – 0.007 | 0.613 |
| *Study arm* |  |  |  |
| Healthy controls | REF | - | - |
| Symptomatic controls | -0.216 | -0.848 – 0.416 | 0.503 |
| Children with TB | 0.207 | -0.446 – 0.862 | 0.534 |
| *Study arm interaction with time (weeks)* | | | |
| Healthy controls | REF | - | - |
| Symptomatic controls | 0.013 | 0.004– 0.021 | 0.003 |
| Children with TB | 0.010 | 0.001 – 0.019 | 0.030 |
| **Abbreviations:** 95% CI = 95% confidence interval, TB = tuberculosis, WAZ = weight-for-age z-score, HAZ = height-for-age z-score | | | |
